# Developing Novel Rice Genotypes Harboring Specific QTL Alleles Associated with High Grain Yield under Water Shortage Stress

**DOI:** 10.3390/plants10102219

**Published:** 2021-10-19

**Authors:** Mohamed Abdelrahman, Mahmoud E. Selim, Mahmoud A. ElSayed, Megahed H. Ammar, Fatma A. Hussein, Neama K. ElKholy, Essam A. ElShamey, Naeem Khan, Kotb A. Attia

**Affiliations:** 1 Rice Research and Training Center, Field Crops Research Institute, Agricultural Research Center, Kafrelsheikh 33717, Egypt; Abdelrahman.rrtc@gmail.com (M.A.); m.selimrrtc@gmail.com (M.E.S.); Mahmoud.rrtc@gmail.com (M.A.E.); ammarrice@gmail.com (M.H.A.); fatma_awad2008@yahoo.com (F.A.H.); neamaelkholy@gmail.com (N.K.E.); 2 Department of Agronomy, Institute of Food and Agricultural Sciences, Florida University, Gainesville, FL 32611, USA; naeemkhan@ufl.edu; 3 Department of Biochemistry, College of Science, King Saud University, P.O. Box 2455, Riyadh 11451, Saudi Arabia

**Keywords:** genetic integration, water stress, Oryza sativa, grain yield, consistent QTL, association analysis, MAS system

## Abstract

Rice is considered a strategic crop for many countries around the world, being the main cash crop for farmers. Water shortage stress occurrence as a result of climate change is among the main threats challenging rice breeders in the last few decades. In the current study, 19 Fn-lines were developed from four populations by crossing a reverse thermo-responsive genic male sterile (rTGMS) line, M.J.5460S, with the three high-quality Egyptian commercial cultivars Giza177, Sakha105, Sakha106 and the promising line GZ7768 as male parents. These newly developed lines, along with their parents, and two water shortage stress-tolerant international genotypes (Azucena and IRAT170), were cultivated under water-shortage stress conditions and compared with their performance under well-watered conditions. Results indicated that the yielding ability of the 19 newly developed lines exceeded those for the two Egyptian parents (Giza177 and Sakha105) under well-watered conditions. The lines M.J5460S/GIZA177-3 and M.J5460S/GIZA177-12 were the best performing genotypes under water shortage stress conditions. The genetic and heritability in broad sense estimates indicated that direct selection for grain yield (GY) under water-shortage stress is highly effective in the current study. Molecular marker analysis revealed that M.J5460S/GIZA177-3 had accumulated the quantitative trait loci (QTL)s, on the chromosomes 2, 3, and 9, which contribute to GY under water-shortage stress from their high yielding tolerant ancestor, M.J5460S. It could be concluded that those lines are high yielding under both well-watered and water-stress conditions harboring several QTLs for yield enhancement under both conditions and that the markers RM555, RM14551, RM3199, RM257, RM242, and RM410 are among the markers that could be used in marker-assisted selection (MAS) breeding programs for such stress condition.

## 1. Introduction

Production of enough food under limited resources of land and water is considered to be the main approach to secure food for those countries facing reduced arable land and water scarcity, especially those undeveloped ones. Securing food with such a method consider a manner for sustaining agriculture and achieving the worldwide sustainable development goals of the 2030 Agenda stated by the United Nations in their 2015 summit. The rice crop is of great importance, being one of the most strategic world commodities and being a staple food for half of the world population [1,2,3], explaining its direct effect on food security. Water scarcity is considered one of the main threats facing global rice production since it requires standing water during the cropping season for the best performance. Accordingly, rice crops are vulnerable to yield reduction if water shortage events occur during the cropping season [4]. Worldwide, 50% or more of the cultivated rice area is projected to suffer from water stress, causing significant losses in rice productivity [5]. An overgrowing population coupled with climate change is yet to increase the negative consequences of water shortages on rice crops. Several countries around the world are considered the most vulnerable to water scarcity and climate change. However, almost half the world’s population will be living in areas of high water stress by 2030 [6]. Egypt, among others, is expected to reach the UN threshold for absolute water scarcity by 2025 [7,8,9], which will make the country struggle to withstand the water shortage and maintain food production.

Continuous attempts are being made to develop drought-tolerant rice genotypes with less dependency on water availability and with enhanced yielding ability under water-scarcity conditions [10]. Grain yield (GY) under water-shortage stress conditions has been proved to be an important criterion of selection for drought tolerance enhancement [11]. Several breeding lines with high yielding under water-shortage stress have been developed by IRRI by adopting this approach [12,13]. Various quantitative trait loci (QTLs) with major effects contributing to GY under water-shortage stress have been reported [14,15,16,17]. These QTLs have been proven to enhance tolerance to water shortage and have been successfully incorporated in different marker-aided breeding programs [18,19]. SSR markers linked to these QTLs have been further employed in the mapping and introgression of such QTLs and others. However, yet this approach has not been applied to introgress and map these QTLs in the Egyptian rice genetic background. Marker-assisted introgression of QTLs, with major effects for GY under water-deficit stress, are considered a fast-track approach for breeding high-yielding water shortage tolerant rice genotypes [20]. Because of these major effects, QTLs should be tested across different genetic backgrounds and across environments to check the consistency of their effect. Furthermore, these identified QTLs are mostly in the background of non-elite genotypes and probably in this case their large effects, will not provide any improvement if introgessed in the newly developed cultivars as the allele may be ubiquitous in the current improved varieties [21].

The use of multi-parent populations has become an increasingly regular procedure in plant genetics and breeding [20]. Therefore, the Egyptian rice genotypes Giza177, Sakha105, Sakha106, and GZ7786, which are elite high-yielding genotypes have been utilized in the current investigation. These rice genotypes were developed for the preferences of the Egyptian rice market. However, they are sensitive to water shortage and yet have specific water requirements leading to high yield losses in case water shortage occurs [3,22]. Improving such genotypes by the introgression of water shortage tolerance will be of great value to increase production and sustain water resources. Accordingly, in the present study, we used the above-mentioned genotypes as male parents for developing several bi-parental populations linked by a water shortage tolerant common parent, M.J.5460S. M.J5460S is an rTGMS line that was found to be high yielding under water- shortage (preliminary unpublished data). rTGMS lines are fertile at high temperatures and sterile at low temperatures [23]. These lines are commonly used for developing two-line hybrid rice seed production. However, in the current investigation, we used this system to develop inbred lines following pedigree breeding. Utilization of marker-assisted selection to identify the genotypes carrying favored GY alleles will increase the accuracy of selecting best performing genotypes. The objective of the current study is to improve the Egyptian cultivars by developing new high yielding lines harboring QTLs contributing to GY under water shortage regime with high yielding ability. To our knowledge, this is the first investigation to develop stable high yielding lines harboring QTLs linked to GY under water-shortage stress using an rTGMS line in the genetic background of Egyptian cultivars.

## 2. Results

### 2.1. The Response of the Newly Developed Lines to Water Shortage Stress

In the results of the current research, a wide range of yield and the component characteristics of the genotypes were recorded. The data was combined for the two seasons 2019 and 2020, and is summarized in Table 1.

The analysis of variance of all traits under study showed highly significant differences among the 26 genotypes (Table 1 and Table 2). It is obvious from the results that the source of variation demonstrated in these traits is due to the characteristics of the tested genotypes under each condition. The overall mean values of the genotypes under normal irrigated condition were 91.86, 103.57, 4.99, 22.7, 1.15, 93.38, and 29.65 for days to heading, plant height (cm), panicle weight (g), panicle length (cm), yield m^−2^ (kg/m^2^), seed set (%), and 1000-grain weight (g), respectively. Apparently, the water shortage stress caused a reduction in the estimates of the overall means for all the studied characters except for the days to heading (91.86 to 101.18 days), explaining the decreased plant growth ability under water shortage. Genotypes with a shorter duration in the field are preferable as their water requirements are considered less than those that reside longer duration in the fields. Among the newly developed lines, M.J5460S/GIZA177-28 (93.93 days) was the best performing genotype under water stress, while M.J5460S/SAKHA106-6 and M.J5460S/GIZA177-3 (88.58 and 88.73 days, respectively) were the best under normal irrigation for days to heading. For panicle weight, several developed lines have higher panicle weight estimates than their corresponding male parents under both normal and water withholding conditions. M.J5460S/GIZA177-3 and M.J5460S/GZ.7768-10 records under drought stress for this trait were comparable to their female parent, M.J5460S and higher than the two drought checks; Azucena and IRAT170. At the same time, the line M.J5460S/GIZA177-28 has the highest records for panicle length under both irrigated and drought conditions (26.57 and 22.13, respectively); these estimates were comparable to the estimates recorded for the water shortage tolerant checks.

The seed set percentage is considered one of the major elements that determine the GY. In this regard, the highest seed set was recorded for the genotypes M.J5460S/GIZA177-12, M.J5460S/SAKHA105-6, and M.J5460S/SAKHA105-15 (95.65, 95.29, and 95.38%, respectively), these estimates are comparable to all the parents and checks estimates under normal irrigated condition. While under the 15-day water withholding stress, the lines M.J5460S/GIZA177-3, M.J5460S/GIZA177-12, and M.J5460S/GZ.7768-10 records (69.28, 67.18, and 67.09%, respectively) were the highest seed set rates among the newly developed lines. Their estimated seed set rate was higher than the male Egyptian (Giza177, Sakha105, Sakha106, and GZ7768) genotypes. Similarly, among the parentage, Sakha105 was the highest for 1000-grain weight, which was estimated at 29.44 g. Several newly developed lines recorded higher than this weight for their 1000-grain weight, among these lines; M.J5460S/SAKHA105-20 recorded the highest estimated 1000-grain weight, 34.22 g. While under water-stress, M.J5460S 1000-grain weight (24.17 g) was the highest among the other parents, also Azucena and IRAT170 recorded the highest estimates in this regard (24.50 and 24.27 g, respectively). Line-3 recorded 24.13 g for its 1000-grain weight, which is not significantly less than the corresponding estimates for M.J5460S and the two drought checks.

**Figure 1:**
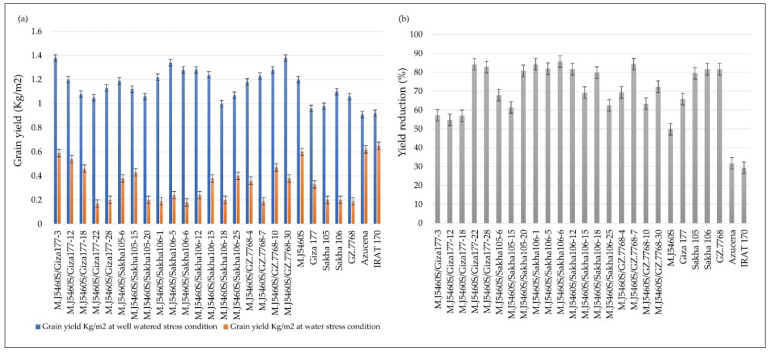
The performance of the newly developed lines. (**a**) GY (kg/m^2^) under well-watered as compared to water-stress conditions and (**b**) yield reduction due to the stress condition.

Table 1 indicates that the selected genotypes have the potential for high yielding ability better than the Egyptian cultivars Giza177 and Sakha105 (0.96 and 0.98 kg/m^2^, respectively) as the lowest GY value under normal irrigated condition was 1.0 kg/m^2^ for the line M.J5460S/SAKHA106-18. At the same time, M.J5460S/GIZA177-3 and M.J5460S/GZ.7768-30 recorded the highest GY values in this regard (1.38 kg/m^2^). While under water-shortage stress, several lines recorded higher values than their corresponding male parents. M.J5460S/GIZA177-3 and M.J5460S/GIZA177-12 (0.59 and 0.54 kg/m^2^) were the highest yielding lines (Figure 1a). Yield reduction under water stress is considered as an indicator to measure the ability of the genotype to withstand such stress. Regarding yield reduction in the newly developed lines due to the exposure of drought stress, Lines M.J5460S/SAKHA106-6, M.J5460S/GZ.7768-7, M.J5460S/GIZA177-22, and M.J5460S/SAKHA106-1 (85.73, 84.33, 84.24, and 84.24%) recorded the highest estimates while lines M.J5460S/GIZA177-3 and M.J5460S/GIZA177-12 were the best as they maintained 57.20 and 54.85% of their yield under water shortage stress (Figure 1b). DSI is an important indicator to measure the susceptibility of the genotypes. In this regard, M.J5460S/GIZA177-3 and L4 (0.57 and 0.55) have the lowest susceptibility index among the newly developed lines.

### 2.2. High Heritability in the Broad Sense under Water-Shortage Stress

The mean performance, genotypic variation, environmental variance, phenotypic variation, genotypic coefficient of variation, phenotypic coefficient of variation, and heritability for the studied characters at normal and stress conditions are presented in Table 3. The genetic variance was estimated for all characters under study and were used to estimate their heritability in the broad sense. The highest heritability estimate was determined for the plant height under normal irrigated conditions (99.18%). These findings, along with those obtained from the partitioning of genetic variance, confirm that the selection might be practiced successfully in late generations.

Among the evaluated characters, panicle weight had the highest genotypic coefficient of variation panicle weight had the highest estimated genotypic coefficient of variation (16.93%) and phenotypic coefficient of variation (9.10%) under normal irrigation, Table 3. Genetic variability analysis indicated that the values of PCV were slightly higher than their corresponding GCV estimates for all studied traits indicating that the selection of the genotypes based on phenotypic data is effective.

### 2.3. Marker Selection Based on Parents’ Genotyping

The five parental genotypes were screened with 28 SSR markers colocalized with QTLs linked to GY under water stress conditions. These GY-contributing QTLs are located on chromosomes 1 (*qDTY1.1*, *qDTY1.2*, and *qDTY1.3*), 2 (*qDTY2.1* and *qDTY2.2*), 3 (*qDTY3.1* and *qDTY3.2*), 9 (*qDTY9.1*) and 12 (*qDTY12.1*) (Table 4). The results of this investigation indicated that among the tested markers, 7 SSR markers were able to discriminate the water-stress withstanding parent M.J. from the Egyptian sensitive genotypes (Supplementary Table S1). These markers are RM555 and RM525 on chromosome 2, which colocalized with *qDTY2.2*. While on chromosome 3, RM14551 colocalized with *qDTY3.1* and RM3199 colocalized with *qDTY3.2*. At the same time, the markers RM257, RM242, and RM410 colocalized with *qDTY9.1* on chromosome 9. The rest of the markers were either monomorphic for the five parents or failed to distinguish the common parent from the other parents.

### 2.4. Marker-Trait Association for GY under Water Stress and Normal Conditions

Subsequent genotyping has been conducted using the identified 7 SSR markers for the 19 newly developed lines (Supplementary Figure S1). The resulted genotypic data was utilized to conduct marker-GY association analysis using SMA. The coefficient of determination R^2^ explaining the marker association with GY and the M.J5460S allele effect is presented in Table 4. Out of the seven polymorphic markers in the current investigation, six markers showed significant association with the generated data under water-shortage stress conditions. On chromosome 2, RM525 marker allele did not exhibit association with GY, while RM555 colocalized with a GY QTL showed a significant association (R^2^ = 17.8) with GY, and the allele effect of this QTL segment showed a 0.12 kg/m^2^ allelic effect. While on chromosome 3, two QTLs showed effect on the phenotypic variation of the GY under water stress. RM14551 and RM3199 segments showed Significant R^2^ values of 25.3 and 15.9 with an allelic effect of 0.17 and 0.11 kg/m^2^, respectively. Interestingly, the markers RM257, RM242, and RM410, which colocalized with the QTL on chromosome 9, exhibited high allelic association with the GY under water shortage. RM242 allele exhibited the highest R^2^ value of 60.3, which increased the GY by about 0.21 kg/m^2^.

### 2.5. Consistent Allele Effect across the Different Water Regimes

The development of such lines was based on the utilization of a common parent with different Egyptian genotypes to enhance their ability under water-stress conditions. This is mainly to make use of the different genetic backgrounds. In order to estimate the consistency of the identified alleles under different water conditions, the SMA was conducted to check the association of those alleles with GY under normal conditions. The results indicated that the allele for RM410, which is colocalized with chromosome 9 GY QTL, showed significant association. Similarly, the allele 14551 showed a significant association and explained 34.7 of the phenotypic variation in GY under water-stress with an allele effect of 0.17 kg/m^2^. Unlike the other markers, those two markers showed consistent effects under both water stress and normal regimes.

## 3. Discussion

Apparently, the main target of plant breeders for developing new lines is to have high-yielding genotypes that could also provide consistent performance against the crop cultivation constrains. Water-stress tolerant lines with high yielding ability under irrigated and water shortage stress conditions are extremely valuable as these lines could be disseminated to different areas and save water. Various efforts have been employed to mitigate water shortage stress in rice by breeding tolerant varieties using conventional breeding supported by MAS. Multiple QTLs with a major effect on GY under water shortage stress have been reported [19]. These QTLs have been incorporated into the genetic background of water-shortage susceptible rice genotypes using the molecular breeding approach [18,24,25,26]. In the current investigation, new lines have been developed by crossing four different Egyptian genotypes with an rTGMS line, and subsequent selections of the lines under normal condition across generation was conducted since the well-watered condition is the major target in rice breeding and, as in most cases, high yielding lines can still give high to moderate yield under drought conditions [17,27] unless the caused reduction was very high.

### 3.1. Phenotypic Response and Lines Performance under Well-Watered and Stress Conditions

It is obvious that the water shortage stress has delayed the flowering time for all the newly developed lines, as it has for their parents and the checks. This symptom was previously observed by [28,29,30]. According to some studies, the delayed flowering under water stress is a good indicator of plant responses to water stress and may explain the plants’ adaptability to tolerate the stress [31]. Early maturing rice genotypes consume less water than long-duration ones [9]. In our study water-shortage stress has negatively affected the performance of the newly developed lines, as well as their corresponding parents. This negative effect was previously reported by [14,22], as the stress caused severe reduction in plant height, biomass, spikelet fertility, and GY. Water shortage stress pushes plants to perform more respiration and reduce photosynthesis, leading to less biomass accumulation and less GY [32,33].

The yield reduction in the newly developed lines ranged from 54.85 to 85.73%, indicating that these lines were subjected to severe water stress. Multiple earlier investigations reported yield reductions of more than 50%, confirming successful water stress screening [3,18]. Poor panicle weight, length, seed set, 1000-grain weight, and seed set are some of the causes of GY reduction under water-stress [32,34]. The current investigation results confirm the ability to use GY under water-shortage as an effective direct selection criterion for enhancing water-shortage stress tolerance in rice. However, the current investigation includes newly developed inbred lines using a common parent, yet the CV and ranges of the studied traits clearly indicate the existence of enough variation for yield and its related traits under both well-watered and water-stressed conditions. This might be due to the utilization of different backgrounds as parentage with the common parent as well as including two water stress tolerant rice checks. Evidently, among the newly developed lines M.J5460S/GIZA177-3, M.J5460S/GIZA177-12, and M.J5460S/GZ7768-10 are good water-shortage tolerant lines that could be used in breeding for water-shortage tolerance enhancement. Those three lines have a GY advantage of 2600, 2100, and 2800 kg/ha over their corresponding Egyptian rice parent.

### 3.2. GY Heritability under Water Shortage Stress Condition

The estimated H^2^ value for GY under water stress was higher than the one under normal conditions and higher than 95%, indicating that direct selection for such traits under water-shortage stress will be effective for improving crop tolerance to water shortage stress. Several previous investigations reported moderate to high H^2^ of GY under similar conditions [31,35]. Furthermore, PH and DTF under water shortage stress recorded high H^2^ estimates in several previous trials [36,37,38]. Beena et al. [39] figured out that the high H^2^ in these traits was mainly owing to the existence of additive gene action and was suitable for direct selection in improving water shortage stress tolerance in rice.

### 3.3. Genotypic Evaluation of the Parents with the Markers Linked to GY QTLs

To date, several QTLs have been reported to contribute to GY under water-shortage stress. These QTLs have been mapped on the different chromosomes and have been successfully used through MAS programs to improve yield production under such environmental conditions using SSR markers [18]. Out of these QTLs, nine previously reported QTLs were investigated in the current study using 28 different SSR markers. The water-shortage stress tolerant genotype rTGMS was used as a common parent to improve four different Egyptian genotypes under water-stress conditions. The utilization of multi-parents was mainly to identify and characterize allelic variants associated with GY that have a continuous contribution to yield increase across different genetic backgrounds simultaneously [40]. Apparently, enough variation exists among parents, which allowed us to identify 7 markers out of 28 that distinguished the common parent water-shortage tolerant allele from the other susceptible parents’ alleles. Among these markers, three markers (RM242, RM257 and RM410) are closely linked to the same QTL on chromosome 9 and 2 markers (RM555 and RM525) are closely linked to the same QTL on chromosome 2. It was speculated that these markers can distinguish the high-yielding tolerant lines from those low-yielding lines. Previously, Venuprasad et al. [15] identified two markers out of 293 markers that can distinguish the high and low phenotypic performance for yield under water-shortage stress.

### 3.4. Marker GY Association Analysis and Common Parent Allele Effect

Single marker analysis was performed using the seven polymorphic markers to test marker-trait association and to identify alleles that could be associated with GY under water-shortage stress. Single maker analysis has been used for QTL analysis and is performed to assess the association between the SMA marker and the target trait [41,42,43]. In the current study, a high association was revealed between the GY under water shortage and the markers RM242, RM257, and RM410 which are closely linked to the QTL on chromosome 9. Swamy et al. [24] reported that this genomic region was introgressed into the genetic background of the IR64 cultivar and showed high phenotypic variance for GY under water shortage stress. Furthermore, the same genomic region was reported to have a QTL for spikelet fertility [44], GY, and plant height under well-watered conditions [45]. Furthermore, our results explained that about 25.1% of the phenotypic variance in GY under water-shortage stress is due to the QTL on chromosome 3, which is closely located to the RM14551 marker. Similarly, RM555 and RM3199 showed significant association with GY under water shortage stress, which is linked to other QTLs on chromosomes 2 and 3, respectively. Those segments were previously reported to harbor validated QTLs for GY under water-shortage stress [18,29], respectively. RM525, which is closely located to the same QTL as RM555, did not exhibit significant association with GY. These results indicated the possibility of using the six primers, which showed significant association with GY under water-stress conditions for genetic discrimination and the MAS program by using these newly developed lines in the breeding for high yielding lines under such stress conditions.

### 3.5. Consistent QTL Effect under Both Stress and Well-Watered Conditions

In our study, it was clear that using lines developed from multiple parents with a common parent resulted in a validated QTL effect across different genetic backgrounds. The effect of those QTLs was further evaluated under the normal irrigated condition in order to identify the QTLs that could be used for yield enhancement under both conditions. Under well-watered conditions, the markers RM14551 and RM410, which are closely linked to the QTLs on chromosomes 2 and 9, respectively, showed significant association with GY. Both primers are very promising to be utilized in the MAS breeding programs for yield enhancement under well-watered or water-stressed conditions.

### 3.6. The Newly Developed Lines Harboring High Effect QTLs

The newly developed lines M.J5460S/GIZA177-3, M.J5460S/GIZA177-12, and M.J5460S/GZ7768-10 are among the best performing genotypes as compared to their corresponding parental genotypes. Those genotypes harboring two or more markers are closely linked to QTLs that have significant contributions to GY under the water-stress conditions. M.J5460S/GIZA177-3, harboring the QTLs on chromosomes 2, 3, and 9, M.J5460S/GIZA177-12, and M.J5460S/GZ7768-10 were developed carrying those QTLs on chromosomes 2 and 9. It is evident that the existence of multiple QTLs in the M.J5460S/GIZA177-3 provided the high yielding ability under both stress and well-watered conditions.

## 4. Materials and Methods

### 4.1. Plant Material Development

**Figure 2:**
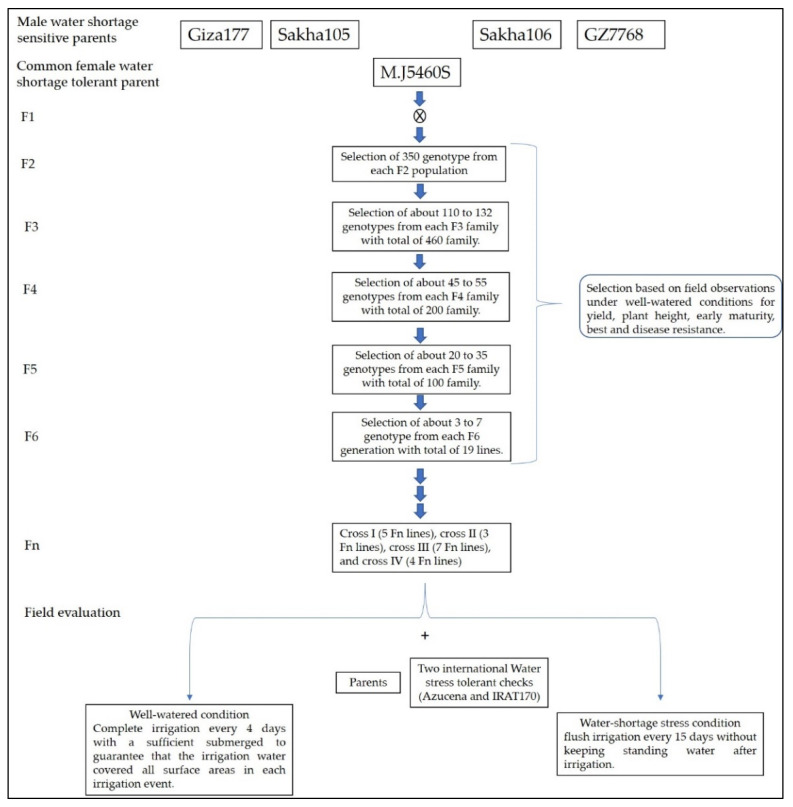
Description of the pedigree breeding scheme by which the new lines were developed and screened under well-watered and water shortage stress conditions.

During two successive summer rice growing seasons of 2019 and 2020, the experimental research trial of the current investigation was carried out at the experimental farm (31.09° N and 30.9° E) and the biotechnology laboratory of the Rice Research and Training Center (RRTC), Sakha, Kafrelsheikh, Egypt. The plant materials consisted of 19 Fn rice lines generated from 4 crosses (I-IV) by crossing a reverse thermo-responsive genic male sterile (rTGMS) line, M.J. 5460s with the three high-quality Egyptian cultivars Giza177, Sakha105, Sakha106, and the promising line GZ7768, as male parents (Table 5). The pedigree selection method was utilized in the F2:Fn segregating generations to reach genotypic stability where cross I (5 Fn genotypes), cross II (3 Fn genotypes), cross III (7 Fn genotypes), and cross IV (4 Fn genotypes) (Figure 2). In particular, the water-shortage stress-tolerant genotypes Azucena and IRAT170 were utilized as checks for the field trials along with the five parental genotypes.

### 4.2. Climate and Soil Properties

The rice crop is cultivated during the summer season in the area under study where the main high temperature with no rain is the common feature of the climate. The maximum temperature is 23–42 °C, the minimum temperature is 12–19 °C, solar radiation is 40–65%, relative humidity is 40–60%, and wind speed is 1.8–6 ms^−1^. The experimental sites were located in the old Nile delta with clayey soil (18.5% sand, 22.5% silt, and 59% clay). This type of soil was found to has a field capacity (42%), available water (22%), wilting point (20%), and lower bulk density (1.2 g cm^−3^) [10].

### 4.3. Field Evaluation Procedures

The 19 Fn genotypes, their parents, and two international water stress-tolerant checks (Azucena and IRAT170) were subjected to both well-watered and water shortage stress. The plant materials were characterized using a randomized complete block design with three replications for the two seasons field evaluation. In the well-watered condition, complete irrigation was induced every four days with a sufficient submerged depth to guarantee that the irrigation water covered all surface areas in each irrigation event. While water shortage stress was induced by flush irrigation every 15 days without keeping standing water after irrigation. The direct dry seeding method was applied for each genotype through plots, where each plot was five one-meter-long rows, 20 cm × 20 cm spacing for each genotype at both normal irrigation and 15-day water-withholding stress. Other cultural practices have been applied in the experimental field as recommended by the national rice research program. The genotypes’ yield and its component characters, as well as the drought susceptibility index (DSI), were determined. The DSI was calculated as DSI = (NS − S)/NS for each genotype, where NS is the yield under irrigated normal conditions and S is the yield under water shortage stress conditions [46]. Genotype performance for days to heading (day), plant height (cm), panicle weight (g), panicle length (cm), yield (kg/m^2^), seed set (%), and 1000-grain weight (g) were assessed for both conditions.

### 4.4. Phenotypic Data Statistical Analysis

The mean phenotypic values for irrigated and water shortage stress at 15-days were utilized for analysis of variance using SAS software, version 9.1 (SAS institute, CARY, NC, USA). Broad-sense heritability (H^2^) for each trait (in each season) was estimated as (H^2^ = genotypic variance/phenotypic variance).

### 4.5. Molecular Analysis

Fresh leaves were collected from the 19 newly developed lines and their corresponding parents individually. The genotypes leaves were exposed to genomic DNA extraction following the procedure of the cetyltrimethylammonium Bromide (CTAB) method [47]. The quantity and quality of the extracted DNA were assayed using the agarose gel electrophoresis-based method using a known concentration of uncut Lambda DNA. The samples were diluted using T_10_E_1_ buffer (10 mM Tris-HCl, 1 mM EDTA, pH 8.0) to reach a final DNA concentration of 20 ng/μL for amplification.

PCR master mix was used following the protocol described by the manufacturer (Simply Biologics, Taoyuan, Taiwan) for the PCR amplification. The PCR was performed in a thermal cycler (SensoQuest Labcycler, Gottingen, Germany) as per the following cycling parameters: initial denaturation at 94 °C for 3 min, followed by 35 cycles of denaturation at 94 °C for 30 sec, annealing at 55 °C for 20 s, and extension at 72 °C for 30 s and final extension at 72 °C for 7 min. Products of the amplified PCR segments were resolved on a 3% Agarose gel using a mini-horizontal electrophoresis system (CBS Scientific, CA, USA) and stained with ethidium bromide (0.5 μg/mL). To detect polymorphism, the gels were visualized and photographed using a gel documentation system (BioDocAnalyze, Biometra). The size of the amplified DNA bands was determined based on the migration relative to a molecular size marker (Bio-Helix, 100 bp DNA ladder).

A set of 28 SSR markers were used for estimating the genetic diversity among parents (Table 2). The original source, repeat motifs, primer sequences, annealing temperature and chromosomal location can be found on the Gramene website (http://www.gramene.org, accessed on 28 November 2020). Markers that had exhibited differential band intensities between the M.J5460S and the other male parents were further utilized for genotyping the 19 developed lines.

### 4.6. Genetic Analysis and Single Marker Analysis (SMA)

Markers that clearly distinguished the female parent from the four male parents were used for genotyping the 19 developed lines along with their corresponding parents. The resulting genotypic data was combined with the phenotypic information to test marker–trait association against the female allele using single marker analysis methods. The SMA was performed using regression analysis [41,43] SPSS Version 17 (SPSS Inc., Chicago, IL, USA) following this model: Y = μ + f (marker) + error. Where Y is equal to the trait value, µ is equal to the population mean, and f (marker) is a function of the molecular marker [48]. Allele effects were estimated as the difference between the averages of all genotypes harboring the female tolerant genotype allele for the respective trait and those carrying the sensitive genotypes alleles (0).

## 5. Conclusions

Water shortage stress coupled with climate change will affect food security all over the world. This scenario is considered a threat to rice cultivation, being sensitive to water shortage, and the occurrence of such stress can cause a significant reduction in rice production. In the current study, genetic improvement of four Egyptian genotypes was conducted to increase yield production under such conditions. New stable lines were developed and screened under water shortage stress. These newly developed lines showed high yielding ability compared to their corresponding Egyptian ancestor genotype. High heritability in the broad sense was recorded under water-shortage stress concluding that direct selection for such a trait in these circumstances will be of great value for the rice breeders. Furthermore, these lines showed a significant GY advantage over their corresponding male parents. Some of the newly developed lines were found to harbor at two or more QTLs linked to GY under water-stress conditions. Out of these QTLs, two were found to have consistent effects under stress and well-watered conditions. These results indicate the success of the approach that was utilized in the current study for the development of new high-yielding lines tolerant to water-stress irrigation.

## Figures and Tables

**Table 1:** Combined mean performance of genotypes for some yield and its component characters.

Characters	Days to Heading		Plant Height (cm)		Panicle Weight (g)		Panicle Length (cm)		Yield (kg/m^2^)		Seed Set (%)		1000-Grain Weight (g)		Drought Susceptibility Index	Reduction %
Genotypes (Fn)	N	S	N	S	N	S	N	S	N	S	N	S	N	S		
M.J5460S/GIZA177-3	88.73	95.26	90.36	75.33	5.83	3.39	20.85	18.06	1.38	0.59	94.51	69.28	30.31	24.13	0.572	57.195
M.J5460S/GIZA177-12	92.06	96.26	101.67	80.33	5.43	2.73	21.30	20.42	1.20	0.54	95.65	67.18	29.42	23.40	0.548	54.849
M.J5460S/GIZA177-18	91.22	96.38	99.70	95.33	4.07	2.76	23.55	17.22	1.08	0.46	94.58	63.14	28.59	23.10	0.570	56.957
M.J5460S/GIZA177-22	93.40	100.93	120.16	76.33	5.21	1.19	23.22	16.62	1.05	0.17	92.76	29.60	30.13	22.17	0.842	84.244
M.J5460S/GIZA177-28	92.09	93.93	113.38	96.67	5.22	1.34	26.57	22.13	1.13	0.20	91.76	38.07	27.39	23.13	0.828	82.764
M.J5460S/SAKHA105-6	90.53	97.93	97.13	85.33	5.72	2.38	25.51	21.71	1.19	0.38	95.29	56.77	31.42	23.40	0.679	67.896
M.J5460S/SAKHA105-15	92.22	103.60	99.09	75.33	5.48	2.32	20.84	15.44	1.12	0.43	95.38	55.49	32.45	21.47	0.614	61.358
M.J5460S/SAKHA105-20	95.15	99.93	98.11	79.67	5.64	1.21	19.00	16.18	1.06	0.20	92.68	27.63	34.22	21.03	0.808	80.784
M.J5460S/SAKHA106-1	90.07	95.93	98.42	79.67	5.63	1.22	21.83	18.16	1.22	0.19	93.41	24.15	32.48	22.33	0.842	84.242
M.J5460S/SAKHA106-5	95.46	103.60	112.74	90.11	5.54	1.40	22.90	21.00	1.34	0.24	93.55	32.39	28.22	23.25	0.819	81.882
M.J5460S/SAKHA106-6	88.58	102.60	86.58	75.33	5.79	1.36	23.09	16.59	1.28	0.18	94.41	21.67	30.13	21.42	0.857	85.725
M.J5460S/SAKHA106-12	98.87	105.15	112.50	73.56	5.59	1.72	22.96	17.12	1.28	0.24	91.61	35.01	29.38	21.77	0.816	81.612
M.J5460S/SAKHA106-15	90.41	110.04	114.76	60.44	5.27	2.30	23.93	16.64	1.24	0.38	91.65	54.08	30.51	23.28	0.692	69.208
M.J5460S/SAKHA106-18	91.73	98.93	87.71	80.11	3.53	1.51	20.30	17.22	1.00	0.20	92.12	18.39	28.36	22.33	0.798	79.821
M.J5460S/SAKHA106-25	95.47	111.93	90.64	75.78	4.65	2.92	18.27	15.40	1.07	0.40	94.49	50.68	26.29	21.20	0.625	62.496
M.J5460S/GZ.7768-4	94.57	102.93	85.73	75.44	5.58	2.28	22.31	19.06	1.18	0.36	92.34	56.60	31.56	22.20	0.693	69.318
M.J5460S/GZ.7768-7	97.07	114.93	92.39	75.67	5.64	1.34	24.30	20.06	1.23	0.19	93.73	28.70	26.79	23.00	0.843	84.328
M.J5460S/GZ.7768-10	95.47	100.93	106.20	80.00	5.53	3.08	20.44	16.36	1.28	0.47	92.07	67.09	28.44	23.20	0.634	63.396
M.J5460S/GZ.7768-30	92.80	102.93	90.84	77.33	5.59	2.69	22.22	19.66	1.38	0.38	93.41	56.12	30.07	24.20	0.724	72.368
M.J5460S	84.00	89.93	95.67	82.33	5.75	3.48	21.86	18.31	1.20	0.60	93.14	72.25	29.05	24.17	0.498	49.847
Giza177	85.71	95.93	102.59	65.33	3.45	1.10	23.54	16.07	0.96	0.33	95.11	61.17	28.71	23.10	0.659	65.892
Sakha105	87.51	94.93	99.94	60.44	3.56	1.24	23.97	16.60	0.98	0.20	93.88	33.07	29.44	23.15	0.795	79.477
Sakha106	88.94	94.93	104.41	73.11	3.48	1.68	24.13	17.11	1.10	0.20	93.25	45.31	26.76	23.87	0.816	81.620
GZ7768	87.86	116.93	105.64	70.44	3.64	1.09	21.07	17.47	1.06	0.19	95.77	31.38	26.98	22.17	0.817	81.681
Azucena	101.64	108.93	158.53	122.56	4.05	3.02	26.06	23.27	0.91	0.62	90.90	74.19	31.86	24.50	0.318	31.783
IRAT 170	86.83	94.93	128.00	110.56	4.89	3.13	27.84	22.48	0.92	0.65	90.49	72.76	31.85	24.27	0.294	29.433
Mean	91.86	101.18	103.57	80.48	4.99	2.07	22.76	18.32	1.15	0.35	93.38	47.78	29.65	22.89	0.69	69.24
LSD	1.98	1.44	1.99	1.67	0.65	0.53	1.09	0.83	0.06	0.01	0.82	0.55	0.73	0.44	-	-

N: well-watered conditions; S: water stress condition; LSD: least significant difference.

**Table 2:** Combined mean square analysis for yield and its component characters.

Characters	Replications (df = 2)		Genotypes (df = 25)		Coefficient of Variation	
	N	S	N	S	N	S
Days to heading	1.637 ^ns^	0.073 ^ns^	51.49 **	141.35 **	0.844	0.406
Plant height (cm)	1.187 ^ns^	0.639 ^ns^	718.86 **	563.00 **	0.756	0.684
Panicle weight (g)	0.123 ^ns^	0.002 ^ns^	2.22 **	1.976 **	1.677	2.704
Panicle length (cm)	0.263 ^ns^	0.184 ^ns^	15.33 **	15.939 **	1.033	0.739
Yield M-2 (kg/m^2^)	0.001 ^ns^	0.001 ^ns^	0.055 **	0.076 **	0.067	0.004
Seed set (%)	0.057 ^ns^	0.012 ^ns^	6.66 **	968.72 **	0.142	0.125
1000-grain weight (g)	0.101 ^ns^	0.041 ^ns^	12.33 **	3.044 **	0.359	0.165

** Statistically significant at 0.01; ^ns^ statistically non-significant. df: degree of freedom; N: well-watered condition; S: water-stress condition.

**Table 3:** Estimate of genetic parameters, heritability in the broad sense, for yield and its component characters under well irrigated and 15 d water stress conditions.

Characters	Mean	Range		GCV (%)	ECV (%)	PCV (%)	H^2^ (%)
		Min	Max				
Well-watered condition							
Days to heading	91.861	84.00	101.64	4.48	1.69	4.78	87.510
Plant height (cm)	103.573	85.73	158.53	14.94	1.35	15.00	99.184
Panicle weight (g)	4.991	3.45	5.83	16.93	9.10	19.22	77.564
Panicle length (cm)	22.765	18.27	27.84	9.85	3.10	10.33	90.989
Yield M-2 (kg/m^2^)	1.149	0.91	1.38	11.73	2.89	12.09	94.269
Seed set (%)	93.383	90.49	95.77	1.58	0.47	1.65	92.015
1000-grain weight (g)	29.648	26.29	34.22	6.81	1.54	6.98	95.147
Water-stress condition							
Days to heading	101.178	89.93	116.93	6.77	0.69	6.81	98.980
Plant height (cm)	80.483	60.44	122.56	17.01	1.36	17.07	99.370
Panicle weight (g)	2.072	1.09	3.48	38.62	11.61	40.33	91.709
Panicle length (cm)	18.321	15.40	23.27	12.53	3.08	12.90	94.287
Yield M-2 (kg/m^2^)	0.347	0.17	0.65	45.81	2.53	45.88	99.695
Seed set (%)	47.776	18.39	74.19	37.61	0.56	37.62	99.978
1000-grain weight (g)	22.891	21.03	24.50	4.37	1.22	4.54	92.729

GCV: genotypic coefficient of variation; ECV: Environmental coefficient of variation; PCV: phenotypic coefficient of variation; H^2^: heritability in the broad sense.

**Table 4:** The polymorphic markers, SMA, and common parent allele effect under well-watered and water-shortage stress conditions.

Marker Allele	QTL	Water Stress Condition		Well Irrigation Condition	
		R^2^	Allele Effect (kg/m^2^)	R^2^	Allele Effect (kg/m^2^)
RM555_240	2.2	17.8 *	0.12	1.1 ^ns^	0.03
RM525_144	2.2	6.2 ^ns^	0.08	0.1 ^ns^	0.01
RM14551_620	3.1	25.3 **	0.17	34.7 **	0.17
RM3199_186	3.2	15.9 *	0.11	0.0 ^ns^	0.00
RM410_195	9.1	27.8 **	0.17	22.5 **	0.13
RM257_166	9.1	31.6 **	0.16	11.8 ^ns^	0.08
RM242_208	9.1	60.3 **	0.21	2.60 ^ns^	0.04

** Statistically significant at 0.01; * Statistically significant at 0.05; ^ns^ Statistically non-significant. QTL: Quantitative Trait Loci, R^2^: coefficient of determination.

**Table 5:** Rice parental genotypes, parentage and origin.

No	Entries	Parents	Origin	Water-Stress Response
1	M.J5460S	rT60-6 MS	China	Tolerant
2	Giza177	[Giza171] Ymji Ni.1//PiNo.4	Egypt	Sensitive
3	Sakha105	GZ5581/GZ4316	Egypt	Sensitive
4	Sakha106	Giza177/Hexi30	Egypt	Sensitive
5	GZ7768	GZ5320/Taninung70	Egypt	Sensitive
6	Azucena	Landrace	Philippines	Tolerant
7	IRAT170	IRAT13/Palawan	Ivory Cost	Tolerant

## Data Availability

All data, tables and figures are original.

## Supplementary Materials

The following are available online at https://www.mdpi.com/article/10.3390/plants10102219/s1, Figure S1: Banding pattern of the sex markers that showed highly significant association with grain yield under water shortage stress. Lane 1: 100 bp ladder; lane 2: M.J56 allele; lanes 3–6 Egyptian parents; Lanes 7: 24 are the newly developed lines alleles. Table S1: Name and loci of markers colocalized with previously reported qDTY.
