# Supplementary material for: Developing Novel Rice Genotypes Harboring Specific QTL Alleles Associated with High Grain Yield under Water Shortage Stress
Source: Plants (Basel). 2021 Oct 19;10(10):2219. doi: 10.3390/plants10102219 (PMC8538742; doi:10.3390/plants10102219)
Supplement: Supplementary file 1 [file plants-10-02219-s001.zip › plants-1408584-supplementary.pdf]

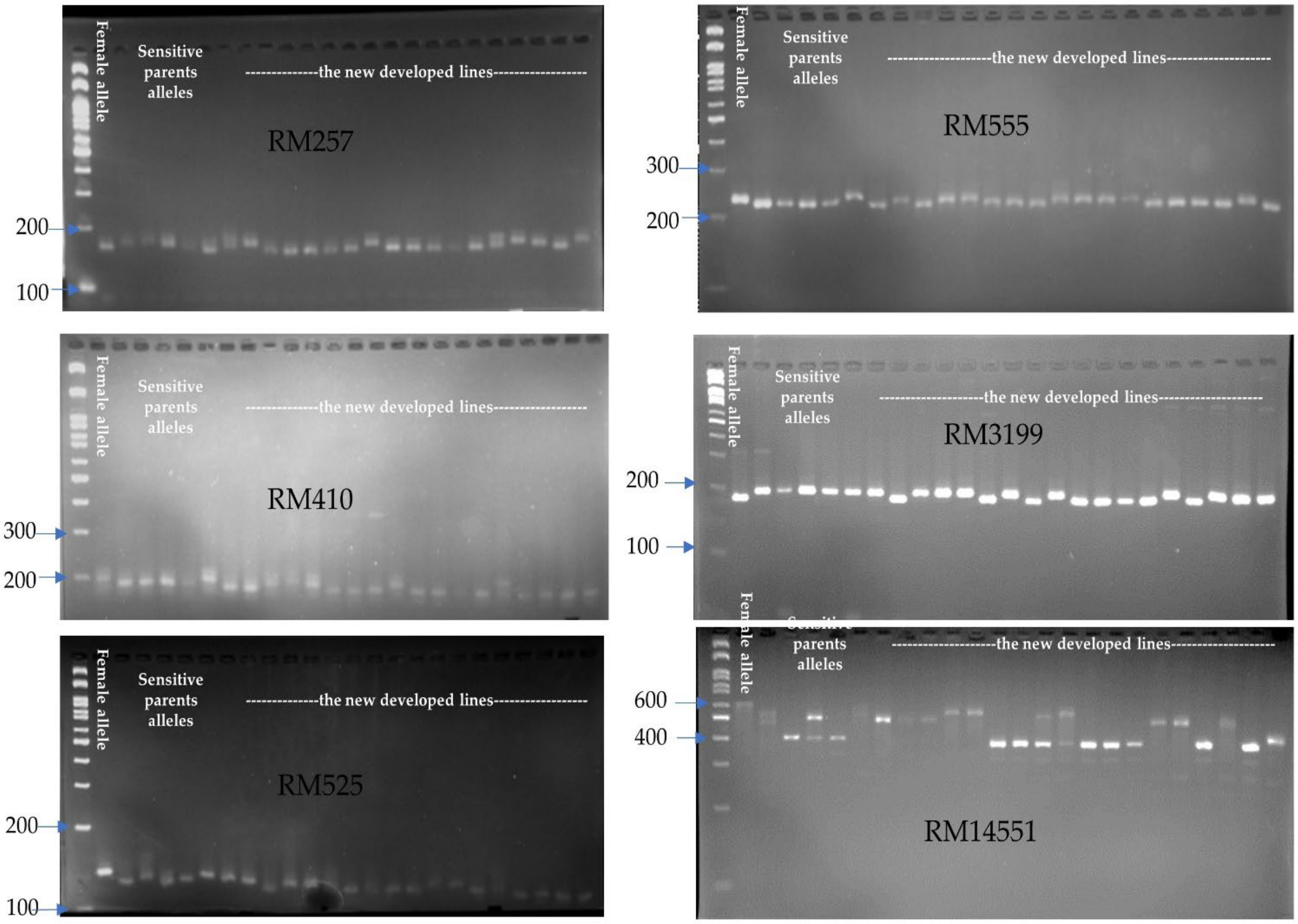

Figure S1. Banding pattern of the sex markers that showed high significant association with grain yield under water shortage stress. Lan 1: 100bp ladder; lane 2: M.J56 allele; lanes 3,4,5,6 Egyptian parents; Lanes 7:24 are the newly developed lines allele.

Table S1. Name and loci of markers co-localized with previously reported qDTY.

**Marker**	**Chromosome No.**	**Locus**		QTL	References
		**cM***	**Kb****		
RM9	1	92.4	23322208	qDTY1.1	[1,2]
RM543	1	145.6	32776801	qDTY1.2	[3]
RM302	1	147.8	32981599		
RM212	1	148.7	33047896		
RM11943	1		37846103	qDTY1.4	[3]
RM431	1	178.3	38888469		
RM3549	2		11388913	qDTY2.1	[4] [5]
RM555	2	59.8		qDTY2.2	[3] [5]
RM263	2	127.5	25889828		
RM525	2	143.7	28292005		
RM166	2	183	.	.	
RM3894	3	3.9	10977	qDTY3.1	[4]
RM14551	3		5402086	5402086	
RM3199	3	137.6	30221208	qDTY3.2	[3]
RM416	3	191.6			
RM566	9	47.7	14651176	qDTY9.1	[3,5]
RM24390	9		15942569		
RM7424	9	60.8	16527147		
RM410	9	64.4	17588741		
RM257	9	66.1	17666088		
RM242	9	73.3	18642201		
RM28048	12	45.2***	14153465	qDTY12.1	[6]
RM28099	12		15896826		
RM511	12	55.5***	17442508		
RM277	12	57.2			
RM1261	12	57.5***	17578154		
RM28166	12		17654663		
RM260	12	61.7			

*Locus according to (www.gramene.org; accessed on 28 November 2020); ** Locus according to [7]; *** Locus according to [6].

References

1. Vikram, P.; Sta Cruz, T.; Espiritu, M.; Valle, M.; Singh, A.; Kumar, A. Major effect QTLs for grain yield under lowland drought stress. In Proceedings of the Poster presented at International Rice Genetics Symposium, 2009; pp. 16-19.

2. Ghimire, K.H.; Quiatchon, L.A.; Vikram, P.; Swamy, B.M.; Dixit, S.; Ahmed, H.; Hernandez, J.E.; Borromeo, T.H.; Kumar, A. Identification and mapping of a QTL (qDTY1. 1) with a consistent effect on grain yield under drought. Field Crops Research 2012, 131, 88-96.

3. Swamy, B.M.; Vikram, P.; Dixit, S.; Ahmed, H.; Kumar, A. Meta-analysis of grain yield QTL identified during agricultural drought in grasses showed consensus. BMC genomics 2011, 12, 1-18.

4. Venuprasad, R.; Dalid, C.; Del Valle, M.; Zhao, D.; Espiritu, M.; Cruz, M.S.; Amante, M.; Kumar, A.; Atlin, G. Identification and characterization of large-effect quantitative trait loci for grain yield under lowland drought stress in rice using bulk-segregant analysis. Theoretical and Applied Genetics 2009, 120, 177-190.
5. Dixit, S.; Swamy, B.M.; Vikram, P.; Bernier, J.; Cruz, M.S.; Amante, M.; Atri, D.; Kumar, A. Increased drought tolerance and wider adaptability of qDTY 12.1 conferred by its interaction with qDTY 2.3 and qDTY 3.2. Mol. Breed. 2012, 30, 1767–1779.
6. Bernier, J.; Kumar, A.; Venuprasad, R.; Spaner, D.; Verulkar, S.; Mandal, N.P.; Sinha, P.K.; Peeraju, P.; Dongre, P.R.; Mahto, R. Characterization of the effect of a QTL for drought resistance in rice, qtl12. 1, over a range of environments in the Philippines and eastern India. Euphytica 2009, 166, 207–217.
7. Project, I.R.G.S. The map-based sequence of the rice genome. Nature 2005, 436, 793–800.
